# Using Different Combinations of Body-Mounted IMU Sensors to Estimate Speed of Horses—A Machine Learning Approach

**DOI:** 10.3390/s21030798

**Published:** 2021-01-26

**Authors:** Hamed Darbandi, Filipe Serra Bragança, Berend Jan van der Zwaag, John Voskamp, Annik Imogen Gmel, Eyrún Halla Haraldsdóttir, Paul Havinga

**Affiliations:** 1 Pervasive Systems Group, Department of Computer Science, University of Twente, 7522 NB Enschede, The Netherlands; b.j.vanderzwaag@utwente.nl (B.J.v.d.Z.); p.j.m.havinga@utwente.nl (P.H.); 2 Department of Clinical Sciences, Faculty of Veterinary Medicine, Utrecht University, 3584 CM Utrecht, The Netherlands; f.m.serrabraganca@uu.nl; 3 Inertia Technology B.V., 7521 AG Enschede, The Netherlands; 4 Rosmark Consultancy, 6733 AA Wekerom, The Netherlands; info@rosmark.nl; 5 Equine Department, Vetsuisse Faculty, University of Zurich, 8057 Zurich, Switzerland; annik.gmel@agroscope.admin.ch (A.I.G.); eyrun.haraldsdottir@uzh.ch (E.H.H.); 6 Agroscope—Swiss National Stud Farm, Les Longs-Prés, 1580 Avenches, Switzerland

**Keywords:** inertial measurement unit, machine learning, breed, gait, feature extraction

## Abstract

Speed is an essential parameter in biomechanical analysis and general locomotion research. It is possible to estimate the speed using global positioning systems (GPS) or inertial measurement units (IMUs). However, GPS requires a consistent signal connection to satellites, and errors accumulate during IMU signals integration. In an attempt to overcome these issues, we have investigated the possibility of estimating the horse speed by developing machine learning (ML) models using the signals from seven body-mounted IMUs. Since motion patterns extracted from IMU signals are different between breeds and gaits, we trained the models based on data from 40 Icelandic and Franches-Montagnes horses during walk, trot, tölt, pace, and canter. In addition, we studied the estimation accuracy between IMU locations on the body (sacrum, withers, head, and limbs). The models were evaluated per gait and were compared between ML algorithms and IMU location. The model yielded the highest estimation accuracy of speed (RMSE = 0.25 m/s) within equine and most of human speed estimation literature. In conclusion, highly accurate horse speed estimation models, independent of IMU(s) location on-body and gait, were developed using ML.

## 1. Introduction

Speed is a key variable to analyze horse locomotion and assess performance [1,2]. Effects of speed on kinematic and kinetic parameters as well as health indicators have been studied in a variety of equine-related areas [3,4,5], including injury prevention during exercise [6], lameness detection [7,8], fitness level evaluation [9,10], and genomics analyses [1,11]. Therefore, equine speed assessment is important as it assists clinicians and equestrians to have a comprehensive view of a horse health and fitness level.

In general, the speed has been estimated by observation and labeling in relative terms (e.g., slow and fast) [12]. Describing locomotion parameters relatively might be influenced by expectation bias [13] and the precision is dependant on the assessor’s expertise [14,15]. As a solution, the speed should be measured quantitatively to become comparable.

There are different horse speed measuring methods using devices in literature. Conventionally, high-speed cameras have been used for this purpose [16,17]. However, a camera should be fixed on a wall or ground, requires scaling, and only captures a limited area. Three-dimensional optical motion capture has also been employed for speed assessment [18]. This method is seen as the ‘gold standard’ for measuring gait kinematics [19]. Although motion capture systems can measure the speed precisely, they are expensive and their use is limited to the laboratory environment [20].

In contrast to the limitations of the above methods, inertial measurement units (IMUs) can quantify movement patterns indoor and outdoor. This technology has been utilized for human and animal motion analysis and is capable of accurately measuring acceleration and angular velocity. Fundamentally, integrating acceleration signal results in velocity. In practice however, the aggregation of errors during the integration process can appear as an accumulated drift from the real value over time [21,22,23,24].

Another portable device for speed measurement is the global positioning system (GPS). This device has been validated for accurate speed measurement [2,25,26,27,28] in human [29] and equine studies [6,11,30,31,32,33,34,35,36]. The advantage of using GPS is portability and low price. However, it cannot be used indoors due to the lack of received signals from satellites. The accuracy might also be affected outdoors if large obstacles hinder the connection.

By using GPS speed as ground-truth and IMUs for portability, estimating horse speed would be feasible. However, IMU data is high dimensional, which complicates the possible connection to one-dimension scalar speed data. Machine learning (ML) can overcome this issue by processing non-linear and high dimensional data to an optimized model [37]. Therefore, this study aims to estimate horse speed using body-attached IMUs and ML methods. To achieve an accurate and complete model, the effects of the number and location of IMUs, ML approaches, gaits, and breeds (with distinct movement patterns) on the estimation model were studied and compared.

## 2. Methods

**Figure 1:**
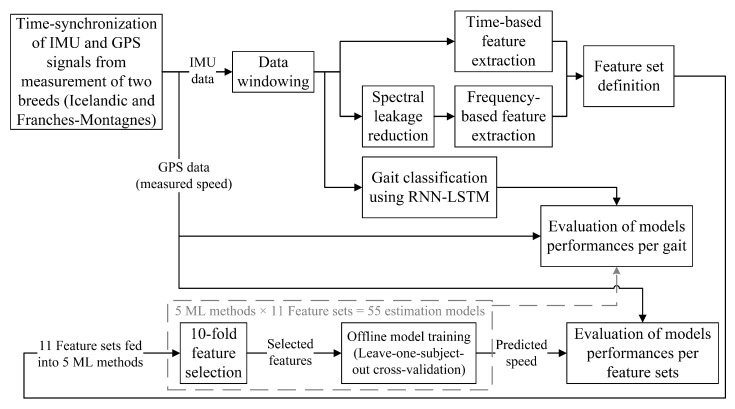
A summary of the model training procedure.

The proposed estimation model is summarized in Figure 1. The body-mounted IMUs and GPS data from two different breeds were collected, time-synchronized, and preprocessed. Subsequently, data was split in windows for Time- and frequency-domain feature extraction. Then, “feature set”s were defined as the extracted features from an IMU or a combination of IMUs. To decrease the number of features used in the estimation model, the optimal group of features was identified using a feature selection method for each feature set. Next, estimation models were trained using popular ML techniques in the literature [38,39], which were support-vector machine (SVM) [40], Gaussian progress regression (GPR) [41], decision tree (DT) [42], boosted trees (BT) [43,44], and random forest [45]. Finally, accuracy of the models were compared.

### 2.1. Data

**Figure 2:**
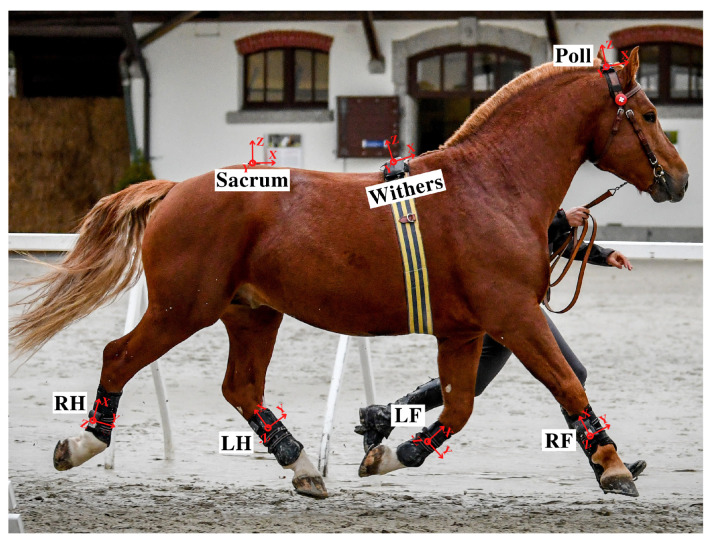
IMUs locations and orientations on horse body. RF: right front limb, LF: left front limb, RH: right hindlimb, LH: left hindlimb. Photographed by Christelle Althaus.

Since horse breeds are biomechanically distinct and present different motion patterns between gaits [46,47,48], we used motion data from two different breeds and various gaits (walk, trot, tölt, pace, and canter) to provide a complete estimation model. The data used in this study was collected from different research projects. In one study, data was collected from fifteen Icelandic horses, ridden at walk, trot, tölt, pace, and canter to study the normal biomechanical properties of different gaits [49]. In another study, twenty-five Franches-Montagnes (FM) horses were walked and trotted in hand to study gaits and phenotype-genotype associations (study not published yet). In both studies, horses were equipped with the same gait analysis system (EquiMoves^®^-www.equimoves.nl) consisting of seven ProMove-mini IMUs (Inertia Technology B.V., Enschede, The Netherlands) (tri-axial accelerometer and tri-axial gyroscope) [50,51] on poll, withers, sacrum, and the lateral aspect of all four limbs (cannon bone). IMU sensors were set to a sampling rate of 200 Hz. Figure 2 demonstrates the IMU locations and orientations on horse body. Horses with known history of lameness or presenting any clear sign of lameness during the measurements were excluded from this study.

The sacrum IMU contained a GPS module (Hornet ORG14xx, OriginGPS Ltd., Airport City, Israel) for the measurement of Icelandic horses, while for FM horses, the GPS module was embedded in the withers IMU. The sacrum and withers were selected for GPS placement since they are the clearest point of the horse’s body for receiving satellites signals. Therefore, the measurement were performed outdoors. The GPS sampling configuration was set to 5 Hz.

The GPS module has been designed to accurately measure the speed (less than 0.01 m/s error for speed value of 30 m/s) using the Doppler shift method [52]. To confirm the accuracy, the module was tested and validated for outdoor speed measurement prior to this study. A detailed description of the validation method and results is provided as Supplementary File S1.

As demonstrated in Figure 2, the three axes of rotation for the sacrum, withers, and poll IMUs were x, y, and z, which were defined in the order as longitudinal axis, mediolateral axis, and vertical axis. For limbs, x, y, and z-axis were aligned to limb (longitudinally), retraction/protraction angle axis, and abduction/adduction angle axis, respectively [50].

### 2.2. Features and Feature Sets

The signals derived from the IMU (x, y, and z axes of acceleration and angular velocity) were low-pass filtered (fourth-order Butterworth filter and 30 Hz cut-off frequency) for noise reduction [50]. Then, the filtered signals were windowed into 256 timesteps windows. The middle of each window was time-synchronized with the corresponding speed data. Since the speed data from GPS were collected every 0.2 s (5 Hz) and the IMU data frequency was 200 Hz, the windows overlapped.

Based on the most chosen features in related literature [53,54,55,56], 23 time and frequency-domain features were selected (Table 1), which later were extracted from the windows. Before the computation of frequency-domain parameters, we used a Hann window to reduce spectral leakage and to enhance the outcome [57].

In total, the dimensionality of the feature vector for each IMU was 23(features)×[3(accelerometersignals)+3(gyroscopesignals)]=138 features. Eleven feature sets were defined from the combination of IMU(s) extracted features (Table 2). The feature sets were defined to compare the accuracy of estimation models in terms of different IMU locations.

### 2.3. Feature Selection

The implementation of a feature selection method has several advantages. It prevents the model from overfitting, decreases training effort, and increases the model performance [55,58]. Therefore, the features of each feature set were used as input for a k-fold sequential Forward floating Selection method (SFFS) [59]. The criteria for adding a new feature in each SFFS step was more than 0.01 point decrease of estimated versus measured speed root mean square error ($RMSE=\sqrt{\sum_{n=1}^{N}\frac{{\left(estimated\;speed\;at\;t_{n}-measured\;speed\;at\;t_{n}\right)}^{2}}{N}}$).

### 2.4. Model Training and Performance Evaluation

To be certain that the dataset from each subject has been used at least one time as training and testing data, all the mentioned ML methods were executed with leave-one-subject-out cross-validation [60]. The estimation models were trained by tuning the hyperparameters and using the selected features in each feature set.

The performance of each model was quantified by calculating the following parameters, mean absolute error (MAE), RMSE, and normalized root mean square error (nRMSE). MAE and nRMSE were defined as follows:

(1) $$MAE=\sum_{n=1}^{N}\frac{estimated\;speed\;at\;t_{n}-measured\;speed\;at\;t_{n}}{N}\;$$

(2) $$nRMSE=\frac{RMSE}{Mean\;of\;measured\;speed\;vector}\;$$

The measured speed was derived from GPS, while the estimated speed was computed using the trained models. For evaluation of the model performance per gait, the signals from All feature set was applied to a gait classification model [61]. The results of the models (11 feature sets × 5 ML techniques = 55 models) are compared in the next section. Matlab R2020a (MathWorks Inc., Natick, MA, USA) was used for all the computations.

## 3. Results

**Figure 3:**
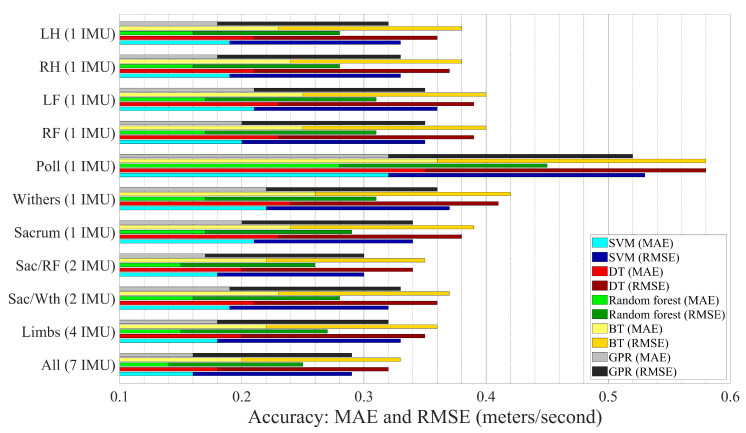
Comparison of models performances using MAE (m/s) and RMSE (m/s).

Approximately 130,000 speed datapoints (26,000 s) have been extracted from the dataset. The estimation performances of the trained models are presented visually in Figure 3 and in details in Table 3. The range of accuracy parameters were 0.14–0.36 m/s for MAE and 0.25–0.58 m/s for RMSE. The performance results of feature sets and methods are compared in the following sections.

### 3.1. Selected Features

According to Table 4, the first-ranked feature of upper body feature sets (sacrum, withers, poll, and Sac/Wth) was extracted from the z-axis of accelerometer signal. The second-ranked feature was also from acceleration signals, which were derived from x and z axes. For the limb feature sets, the z-axis of angular velocity signal ranked the highest. Moreover, the second-ranked feature for both front limbs feature sets were obtained from x-axis of acceleration signal, while for both hindlimbs and Limbs feature sets were selected from z-axis of gyroscope output. Furthermore, all the selected features of limbs feature set were from z-axis of angular velocity.

### 3.2. Feature Set

It can be inferred from Figure 3 and Table 3 that All feature set provided the best performance (RMSE = 0.25–0.33 m/s). In addition, the feature sets from both hindlimbs IMU signals were labeled as the lowest RMSE and MAE among the individual IMU feature sets in estimating the speed (RMSE = 0.28 and MAE = 0.16 m/s). It should be noted that the accuracy of models except the model trained with poll features (RMSE = 0.45–0.58 m/s) were approximately as high as the model based on All feature set.

### 3.3. Machine Learning Technique

Similar to the performance comparison of the feature sets, the differences between performances of models based on SVM, GPR, and random forest methods were insignificant. Nonetheless, considering the small differences, the best ML method in terms of accuracy was random forest. Conversely, models based on DT and BT methods had the worst performance among the ML techniques used in this study.

### 3.4. Gait

As shown in Table 5, the RMSE and nRMSE increased and decreased respectively as the model estimated the faster gaits. The lowest RMSE (0.20 m/s) was obtained during walk, which was lower than the total RMSE (0.25 m/s). During pace, the model was indicated the lowest nRMSE (4.12%) among other gaits.

## 4. Discussion

The main goal of this paper was to develop an accurate model for estimating the speed of horses using IMUs. The derived speed from GPS was used as ground-truth to compare the performances of models trained on five ML methods. Each ML method was trained on eleven feature sets to compare the estimation accuracy in terms of the number and placement of IMUs on the horse body. The feature sets consisted of time- and frequency-domain features extracted from the output of seven IMUs, which was an extensive database of acceleration and angular velocity signals.

The selected features from the angular velocity signal in limb-related feature sets (Table 4) can be explained by the fact that equine limbs move in reciprocating motion. This motion influences the stride frequency, and stride frequency amplifies the locomotion speed. Therefore, a feature extracted from rotation around the z-axis of limb IMU can be an indicator of change in stride frequency [62,63,64]. In addition, the sacrum, withers, and poll feature sets had z-axis acceleration signal extracted features as their highest rank features. The explanation can be similar to the limbs since by using vertical acceleration of the mentioned IMUs, it is possible to determine the periodical pattern of their vertical displacements. Thus, the vertical displacement is affected by the hoof-on and hoof-off rate, i.e., stride frequency [50,65].

**Figure 4:**
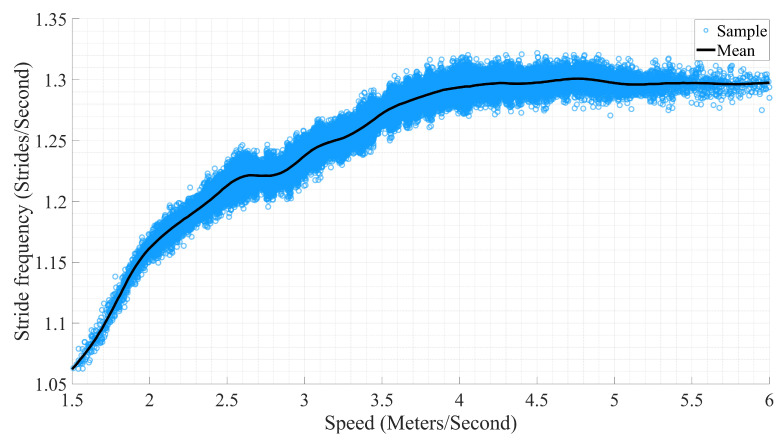
Stride frequency versus speed.

To analyze the relation of stride frequency and speed in the dataset, hoof-on moments of the right front limb were detected using a validated method from another study [65]. Then, stride frequency was calculated using the timing of detected hoof-ons, time-synchronized with speed values. According to the result shown in Figure 4, as speed increases, the stride frequency also increases. To investigate the possibility of estimating speed using stride frequency, we fitted second- and third-degree polynomials to the data and computed the accuracy. However, the accuracy was low compared to the results of the ML models (2nd-degree polynomial: MAE = 0.51 m/s, RMSE = 0.8 m/s and $R^{2}$ = 0.74; 3rd-degree polynomial: MAE = 0.52 m/s, RMSE = 1.36 m/s, and $R^{2}$ = 0.73), which is concurrent with the outcome of other studies [66,67]. Therefore, it requires a more complex model to estimate speed accurately, which has been done successfully in this study.

The performance of all the models per gait had a similar pattern in the means of RMSE and nRMSE. To present this pattern, the performance of the model based on All feature set was demonstrated in Table 5. As the horses switched to faster gaits- from walking to cantering- the RMSE of models increased while their nRMSE slightly decreased. It should be noted that RMSE indicates the actual differences from the mean while nRMSE shows the differences relative to mean. Therefore, these alteration suggests that the model maintains its high accuracy on different speeds regardless of the gait.

Random forest, GPR, and SVM models obtained better results compared to DT and BT. Moreover, the performance of models trained with random forest algorithms was the best and the training and testing duration were significantly shorter compared to the models based on SVM and GPR. Therefore, this method not only has an advantage in accuracy but also reduces computational time.

The model in this study is more accurate than the only other speed estimation model in equine literature (average RMSE = 0.43 m/s) [68]. Moreover, our model is able to estimate the speed of two distinct breeds and five gaits by attaching an IMU to one of the seven body locations in contrast to the mentioned model, which estimates the speed of one breed during canter by attaching an IMU to the saddle. In addition, five ML techniques were compared for choosing the best performing while only SVM was used in [68]. Therefore, our model is the most complete in terms of accuracy, IMU placement options, and different motion patterns (breed and gait) support in the equine literature.

Despite the scarcity of speed estimation studies in equine literature, various studies investigated human speed estimation using IMU. For instance, linear regression was applied to the output of a single IMU to estimate older adults’ walking speed, where the model yielded RMSE ≈ 0.069 m/s accuracy [69]. Another study also developed a walking speed estimation model using GPR, which achieved an accuracy (RMSE) of 0.066–0.095 m/s [70]. By comparing the results of the present model and the mentioned studies, we can conclude that our model is more general than the models from human studies in terms of multiple motion patterns support and different IMU placements, while others focused only on estimating the walking speed [24]. As shown in Table 5, the nRMSE range of the model was 4.12–11.76%. By calculating the nRMSE range of [70] (5.4–10%) and [69] (6.6–14.7%), it can be derived that the model in this study can estimate the speed more accurately than aforementioned studies.

It is more attractive to predict the speed with only one sensor since it is cheaper and the deployment on a subject’s body is simpler. However, according to the results, the model based on Sacrum/RF feature set showed better accuracy than models based on single IMU. During the training and competition, the sacrum IMU may fall off, since it is attached to the horse with double-sided tape without a strap. Therefore, it might limit the usability of the sacrum-based models to research purposes. In contrast, the limbs IMUs can be fixed around the tendon boots. Finally, the accuracy differences between the Sacrum/RF model and individual limb-based models (especially hindlimbs) are negligible. Considering the easier deployment, using only one limb IMU for estimation is nearly as accurate while being more applicable during training and competitions.

## 5. Conclusions

In this study, three novel outcomes were achieved. First, our investigation for the optimal IMU placement concluded that placing only one IMU on any limb, withers, or sacrum will result in high speed estimation accuracy. Second, our model can estimate the speed during distinct motion patterns from two different breeds and five gaits. Finally, the performances of five prevalent ML techniques on estimating the horse speed were compared.

In total, 55 ML estimation models were trained, validated, and evaluated. Models based on random forest method performed better than SVM, GPR, DT, and BT methods. Moreover, the model can accurately estimate the speed during all gaits using the output signals of only one IMU (limb, sacrum, or withers). Therefore, this robust model offers flexibility to researchers, equestrians, and breeders in terms of IMU location on the horse’s body, and thus, they can benefit from accurate speed estimation in training and competition. In future studies, model capability might be explored by adding data from different breeds or age-specific horses measurements. Performance of the model can also be investigated by training it on novel deep learning methods.

## Figures and Tables

**Table 1:** Time- and frequency-domain features.

Time-Domain Feature	Equation
Maximum	*max = Maximum value of the window*
Minimum	*min = Minimum value of the window*
Mean ($\bar{x}$)	$$mean=\frac{1}{N}\sum_{n=1}^{N}x_{n}$$
Median	*mdn = Median value of the window*
Standard deviation (σ)	$sd=\frac{1}{N-1}\sum_{n=1}^{N}{\left(x_{n}-\bar{x}\right)}^{2}$
First quartile	*p25 = 25th percentile of the window*
Third quartile	*p75 = 75th percentile of the window*
Kurtosis	$krt=\frac{1}{N}\sum_{n=1}^{N}{\left(\frac{x_{n}-\bar{x}}{\sigma }\right)}^{4}$
Skewness	$skw=\frac{1}{N}\sum_{n=1}^{N}{\left(\frac{x_{n}-\bar{x}}{\sigma }\right)}^{3}$
**Frequency-domain Feature**	
Spectral entropy	$ent=-\sum_{n=1}^{N}(\frac{|x\left(\omega_{i}\right){|}^{2}}{\sum_{n=1}^{N}{\left|x\left(\omega_{i}\right)\right|}^{2}}\times \ln\frac{|x\left(\omega_{i}\right){|}^{2}}{\sum_{n=1}^{N}{\left|x\left(\omega \right)\right|}^{2}})$
Spectral energy	$enrg=\sum_{n=1}^{N}{\left|x\left(\omega \right)\right|}^{2}$
Magnitude of Fourier transform 1st six coefficients	$fft_{k}=Magnitude\left(\sum_{n=0}^{N-1}x_{n}e^{-j(\frac{2\pi }{N})kn}\right)$
Phase angle of Fourier transform 1st six coefficients	$ang_{k}=arctan\left(\frac{\sum_{n=0}^{N-1}x_{n}sin(\frac{2\pi }{N})kn}{\sum_{n=0}^{N-1}x_{n}cos(\frac{2\pi }{N})kn}\right)$

N= *Window size,* $x_{n}\in$ *Window,* k= *Fourier transform coefficient number (1, 2, …, 6),* $\omega =\sum_{n=0}^{N-1}x_{n}e^{-j\left(\frac{2\pi }{N}\right)\left(N-1\right)n}$

**Table 2:** Feature sets definition.

Feature Set	IMU Positions						
	Sacrum	Withers	Poll	RF	LF	RH	LH
All	×	×	×	×	×	×	×
Limbs				×	×	×	×
Sac/Wth	×	×					
Sac/RF	×			×			
Sacrum	×						
Withers		×					
Poll			×				
RF				×			
LF					×		
RH						×	
LH							×

*RF: Right front limb, LF: Left front limb, RH: Right hindlimb, LH: Left hindlimb.*

**Table 3:** Accuracy of the models- MAE(m/s) and RMSE (m/s).

Feature Set	SVM		DT		Random Forest		BT		GPR	
	MAE	RMSE	MAE	RMSE	MAE	RMSE	MAE	RMSE	MAE	RMSE
All	0.16	0.29	0.18	0.32	0.14	0.25	0.20	0.33	0.16	0.29
Limbs	0.18	0.33	0.20	0.35	0.15	0.27	0.22	0.36	0.18	0.32
Sac/Wth	0.19	0.32	0.21	0.36	0.16	0.28	0.23	0.37	0.19	0.33
Sac/RF	0.18	0.30	0.20	0.34	0.15	0.26	0.22	0.35	0.17	0.30
Sacrum	0.21	0.34	0.23	0.38	0.17	0.29	0.24	0.39	0.20	0.34
Withers	0.22	0.37	0.24	0.41	0.17	0.31	0.26	0.42	0.22	0.36
Poll	0.32	0.53	0.35	0.58	0.28	0.45	0.36	0.58	0.32	0.52
RF	0.20	0.35	0.23	0.39	0.17	0.31	0.25	0.40	0.20	0.35
LF	0.21	0.36	0.23	0.39	0.17	0.31	0.25	0.40	0.21	0.35
RH	0.19	0.33	0.21	0.37	0.16	0.28	0.24	0.38	0.18	0.33
LH	0.19	0.33	0.21	0.36	0.16	0.28	0.23	0.38	0.18	0.32

*RF: Right front limb, LF: Left front limb, RH: Right hindlimb, LH: Left hindlimb.* A gradient of color (from dark blue to dark red) was chosen to highlight the accuracy of each model. Dark blue indicates the highest accuracy (lowest RMSE and MAE) while dark red presents the lowest accuracy (highest RMSE and MAE).

**Table 4:** The highest ranked (first to sixth) selected features using SFFS based on random forest method.

Feature Set	#1	#2	#3	#4	#5	#6
All	max_gyr${}_{z}$	sd_acc${}_{z}$	mean_gyro${}_{z}$	p75_gyr${}_{z}$	fft${}_{1}$_acc${}_{x}$	max_acc${}_{x}$
	(LH)	(sacrum)	(sacrum)	(LF)	(withers)	(sacrum)
Limbs	max_gyr${}_{z}$	p75_gyr${}_{z}$	p25_gyr${}_{z}$	min_gyr${}_{z}$	p75_gyr${}_{z}$	p25_gyr${}_{z}$
	(LH)	(LF)	(LH)	(RH)	(RH)	(RF)
Sac/Wth	sd_acc${}_{z}$	sd_acc${}_{z}$	ent_gyr${}_{z}$	p75_acc${}_{x}$	ent_acc${}_{z}$	skw_acc${}_{z}$
	(withers)	(sacrum)	(sacrum)	(withers)	(sacrum)	(sacrum)
Sac/RF	p25_gyr${}_{z}$	sd_acc${}_{z}$	max_acc${}_{x}$	mean_acc${}_{x}$	sd_gyr${}_{y}$	sd_gyr${}_{z}$
	(RF)	(sacrum)	(RF)	(sacrum)	(sacrum)	(RF)
Sacrum	min_acc${}_{z}$	sd_acc${}_{z}$	ent_gyr${}_{z}$	sd_gyr${}_{x}$	sd_gyr${}_{y}$	ent_acc${}_{z}$
Withers	sd_acc${}_{z}$	p75_acc${}_{x}$	sd_acc${}_{x}$	mean_gyr${}_{z}$	ent_acc${}_{z}$	sd_gyr${}_{z}$
Poll	fft${}_{3}$_acc${}_{z}$	sd_acc${}_{x}$	sd_gyr${}_{y}$	ent_acc${}_{z}$	krt_acc${}_{z}$	ent_acc${}_{x}$
RF	p25_gyr${}_{z}$	max_acc${}_{x}$	sd_gyr${}_{z}$	mdn_gyr${}_{z}$	sd_gyr${}_{y}$	sd_gyr${}_{x}$
LF	p75_gyr${}_{z}$	max_acc${}_{x}$	sd_gyr${}_{z}$	sd_acc${}_{y}$	sd_gyr${}_{y}$	mdn_gyr${}_{z}$
RH	min_gyr${}_{z}$	p75_gyr${}_{z}$	mdn_acc${}_{x}$	min_acc${}_{x}$	mean_gyr${}_{x}$	p75_acc${}_{x}$
LH	max_gyr${}_{z}$	p25_gyr${}_{z}$	p75_acc${}_{x}$	mean_gyr${}_{x}$	min_acc${}_{x}$	p75_acc${}_{y}$

*RF: Right front limb, LF: Left front limb, RH: Right hindlimb, LH: Left hindlimb; gyr_x,y,z_: x, y, and z axis of angular velocity signal.*

**Table 5:** Performance of model (based on “All” feature set and Random forest method) per gait type.

Gait	Measured-	Predicted Speed Error	
	Speed (±SD) ($\frac{m}{s}$)	RMSE ($\frac{m}{s}$)	nRMSE
All	3.25 (±1.63)	0.25	7.69%
Walk	1.70 (±0.17)	0.20	11.76%
Trot	3.30 (±0.23)	0.31	10.03%
Tölt	3.90 (±0.23)	0.28	7.18%
Canter	4.95 (±0.43)	0.34	6.87%
Pace	7.52 (±1.43)	0.31	4.12%

## Data Availability

The data that support the findings of this study are available from the corresponding authors upon reasonable request.

## Supplementary Materials

The following are available online at https://www.mdpi.com/1424-8220/21/3/798/s1, Supplementary File S1.
