# Supplementary material for: Using Different Combinations of Body-Mounted IMU Sensors to Estimate Speed of Horses—A Machine Learning Approach
Source: Sensors (Basel). 2021 Jan 26;21(3):798. doi: 10.3390/s21030798 (PMC7865839; doi:10.3390/s21030798)
Supplement: Supplementary file 1 [file sensors-21-00798-s001.pdf]

GPS Validation study:

Introduction:

The GPS device used in this study had not been validated in scientific papers. Thus, a validation study was proposed prior to the main study to evaluate the sensor accuracy in speed calculation. Afterward, the speed data from the GPS sensor was exploited as ground-truth for building the speed estimation model using IMU data and machine learning algorithms.

Method:

An implanted GPS sensor (ORG141x, OriginGPS Ltd) into an IMU (ProMove-mini, Inertia Technology B.V., Enschede, the Netherlands) was selected for this study. It is capable of outputting data in 1 and 5 Hz frequencies. In order to have a near-instant velocity (average velocity on a very short interval), 5 Hz frequency was selected for the experiment. In other words, the GPS output a speed datapoint every 0.2 seconds using the Doppler effect technique.

To validate the GPS device for measuring velocity, a ground truth with the same frequency was required. High-speed cameras have been used in a number of studies for horse speed measurement [1-2]. GoPro Hero 5 high-speed camera (GoPro, Inc., San Mateo, CA, USA) was selected for this purpose, which has been used for scientific purposes in multiple studies. This camera is capable of recording video in 240 frames per second, which is higher than the GPS frequencies. In this study, the camera was set to record in 240 fps and linear mode (preventing the video footage from curving).

A straight track with no buildings and trees around was chosen for the experiment. To provide a measurement reference, the path was marked with two stakes (60 cm in height) three meters apart and nine sticks (50 cm in height and every 30 cm) in between. The camera was placed on a tripod five meters from the middle stick and 5.22 meters from the stakes. The sticks and the camera stayed in the same position during all the trials.

A bicycle was equipped with the GPS sensor. The sensor was firmly fixed on bicycle headtube by an elastic Velcro strap and it was connected to a laptop via Bluetooth. The output data was stored in real-time using a custom software. During the trials, the bicycle was ridden closely behind the sticks while it was recorded with the camera. The experiment was done with three speeds (1.5, 4.5, and 7.5 m/s), each 150 trials.

After the experiment, the video footage and the GPS data were time synchronized. The second datapoint (during the time that the bicycle was between the two sticks) was selected as the GPS speed for each trial. In addition, the speed from the recorded videos was calculated as the distance between the first and second GPS speed occurrence (±1 cm) divided by 0.2 sec. Finally, the speed estimation error of GPS in comparison to video was analyzed using mean absolute error (MAE) and mean absolute percentage error (MAPE) defined as below:

$$MAE = \sum_{n-1}^{N} \frac{estimated\ speed\ at\ t_n - measured\ speed\ at\ t_n}{N}$$

$$MAPE = \sum_{n-1}^{N} \left| \frac{estimated\ speed\ at\ t_n - measured\ speed\ at\ t_n}{N \times measured\ speed\ at\ t_n} \right|$$

Results and discussion:

A total of 450 speed values (range between 1.5 to 7.5 m/s) were collected using the GPS sensor. According to Table 1, in the higher speeds, the error is higher as well. In contrast, the percentage of mean absolute error is lower in fast speed. In total, the errors in all speeds are small enough for practical purposes.

The GPS mean and range of MAPE in measuring slow speed was higher and wider than faster velocities. The reason is relevant to the accuracy of video footage and the camera, which was the limitation of the current study. The distance measurement accuracy with the recorded video was ±1 cm, and time duration was always 0.2 s. The calculation yielded an accuracy of ±0.05 m/s. This amount of precision in low speed might lead to higher MAPE than faster speeds. This is also true by considering the lower MAE in 1.5 m/s lower than 4.5 and 7.5 m/s. In total, the errors in none of the speed values were significant, which means this GPS is accurate in measuring instant velocity in the range of different types of horse gait as same as different speed values [3].

Table 1- GPS validation results

Speed	Mean (SD*) (m/s)		MAE (SD) (m/s)	MAPE (SD) (%)
	GPS	Camera		
Slow (1.5 m/s)	1.71 (0.17)	1.71 (0.16)	0.03 (0.02)	1.85 (1.41)
Medium (4.5 m/s)	4.35 (0.21)	4.37 (0.23)	0.07 (0.03)	1.67 (0.8)
Fast (7.5 m/s)	7.49 (0.26)	7.49 (0.26)	0.09 (0.05)	1.26(0.7)

* Standard deviation

References:

1- Fredricson, I.; Drevemo, S.; Dalin, G.; Hjerten, G.; Björne, K. The application of high-speed cinematography for the quantitative analysis of equine locomotion. Equine Veterinary Journal 1980, 12, 54–59. doi:10.1111/j.2042-3306.1980.tb02309.x.

2- Ratzlaff, M.H.; Shindell, R.M.; White, K.K. The interrelationships of stride lengths and stride times to velocities of galloping horses. Journal of Equine Veterinary Science 1985, 5, 279–283. doi:10.1016/S0737-0806(85)80064-2.

3- Robilliard, J.J.; Pfau, T.; Wilson, A.M. Gait characterisation and classification in horses. Journal of Experimental Biology 2007, 210, 187–197. doi:10.1242/jeb.02611.
